# Supplementary material for: Simple In-House Fabrication of Microwells for Generating Uniform Hepatic Multicellular Cancer Aggregates and Discovering Novel Therapeutics
Source: Materials (Basel). 2019 Oct 11;12(20):3308. doi: 10.3390/ma12203308 (PMC6829256; doi:10.3390/ma12203308)
Supplement: Supplementary file 1 [file materials-12-03308-s001.pdf]

materials

Supplementary Information

Simple In-House Fabrication of Microwells for Generating Uniform Hepatic Multicellular Cancer Aggregates and Discovering Novel Therapeutics

Chiao-Yi Chiu [1], Ying-Chi Chen [1], Kuang-Wei Wu [1], Wen-Chien Hsu [1], Hong-Ping Lin [2], Hsien-Chang Chang [1,3,4], Yung-Chun Lee [4,5], Yang-Kao Wang [6] and Ting-Yuan Tu [1,3,4,*]

1 Department of Biomedical Engineering, National Cheng Kung University, Tainan 70101, Taiwan; judyq0401@gmail.com (C.-Y.C); yingtzai@gmail.com (Y.-C.C.); stephen0001345@gmail.com (K.–W.W.); d2532875@gmail.com (W.–C.H.); hcchang@mail.ncku.edu.tw (H.–P.C.)
2 Department of Chemistry, National Cheng Kung University, Tainan 70101, Taiwan; hplin@mail.ncku.edu.tw
3 Medical Device Innovation Center, National Cheng Kung University, Tainan 70101, Taiwan
4 Center for Micro/Nano Technology Research, National Cheng Kung University, Tainan 70101, Taiwan
5 Department of Mechanical Engineering, National Cheng Kung University, Tainan 70101, Taiwan; yunglee@mail.ncku.edu.tw
6 Department of Cell Biology and Anatomy, College of Medicine, National Cheng Kung University, Tainan 70101, Taiwan; humwang@mail.ncku.edu.tw
* Correspondence: tingyuan@mail.ncku.edu.tw

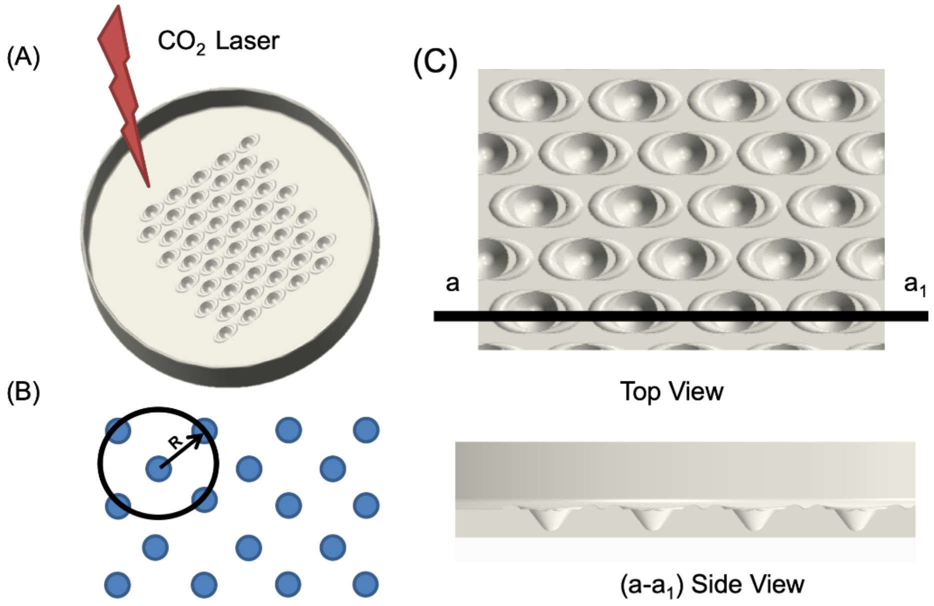

Figure S1. Schematic of microwell prototyping. (**A**) Cell culture plates were ablated by CO_2 laser to create microwell structures on the substrate; (**B**) Staggered microwell arrangement; (**C**) Illustration of the top and side views of the concave microwells and recast region due to laser ablation.

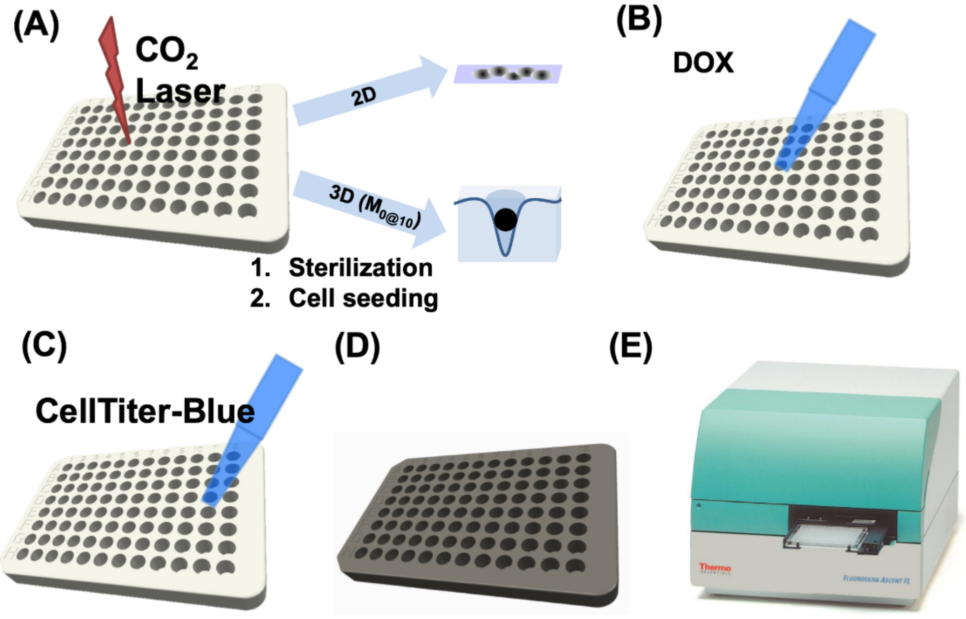

Figure S2. Schematic diagram of a 96-well plate prototype with microwells for the comparison with 2D MCAs and drug screening. (**A**) The prototype of size-controlled microwells in a 96-well multi-well plate was generated using CO_2 laser ablation; (**B**) MCAs are formed in four days, at which time, DOX was added at a range of concentrations to each well of the 96-well plate and incubated for 12 h; (**C**) On the fifth day, the supernatant was aspirated and 20 μL/well of CellTiter-Blue Reagent and medium were added at the appropriate levels such that the final volume of each well was 100 μL; (**D**) The supernatant with CellTiter-Blue was transferred to an opaque 96-well plate to minimize background fluorescence; (**E**) Fluorescence was recorded by Luminoskan Ascent at excitation/emission wavelengths of 560/590 nm.

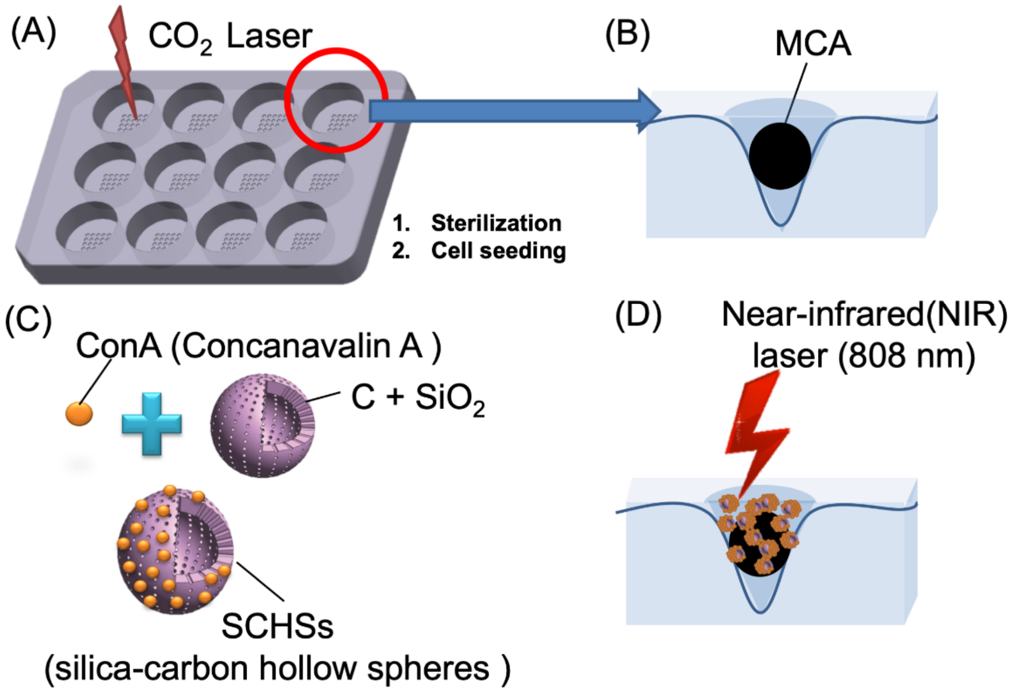

Figure S3. Formation of MCAs combined with ConA-conjugated SCHSs for photothermal treatment. (**A**) MCAs are formed in a 12-well plate with prototype microwells; (**B**) Illustration of an MCA formed in a microwell ($M_{0@10}$); (**C**) A schematic diagram of ConA-conjugated SCHSs is shown; (**D**) ConA-conjugated SCHSs are associated with MCAs and exposed to a near-infrared laser.

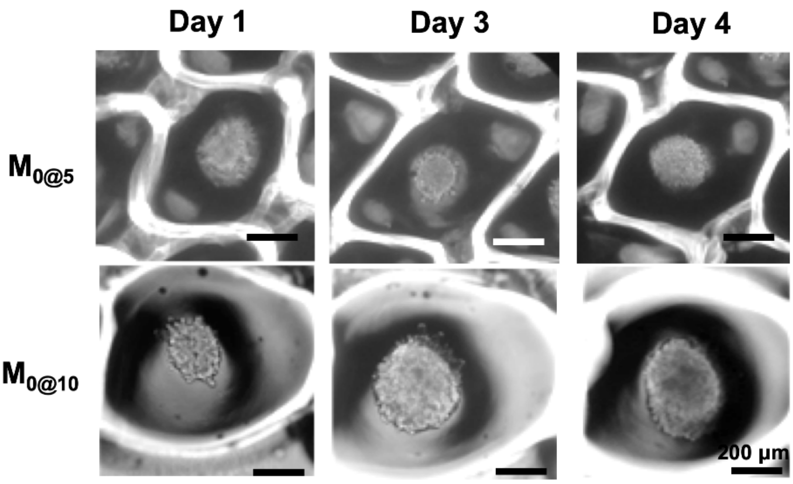

Figure S4. Formation of hepatic MCAs in different parametric microwells. Cell cultures are shown 1, 3, and 5 days after cell seeding in microwells fabricated by laser power of 5 W ($M_{0@5}$) and 10 W ($M_{0@10}$).

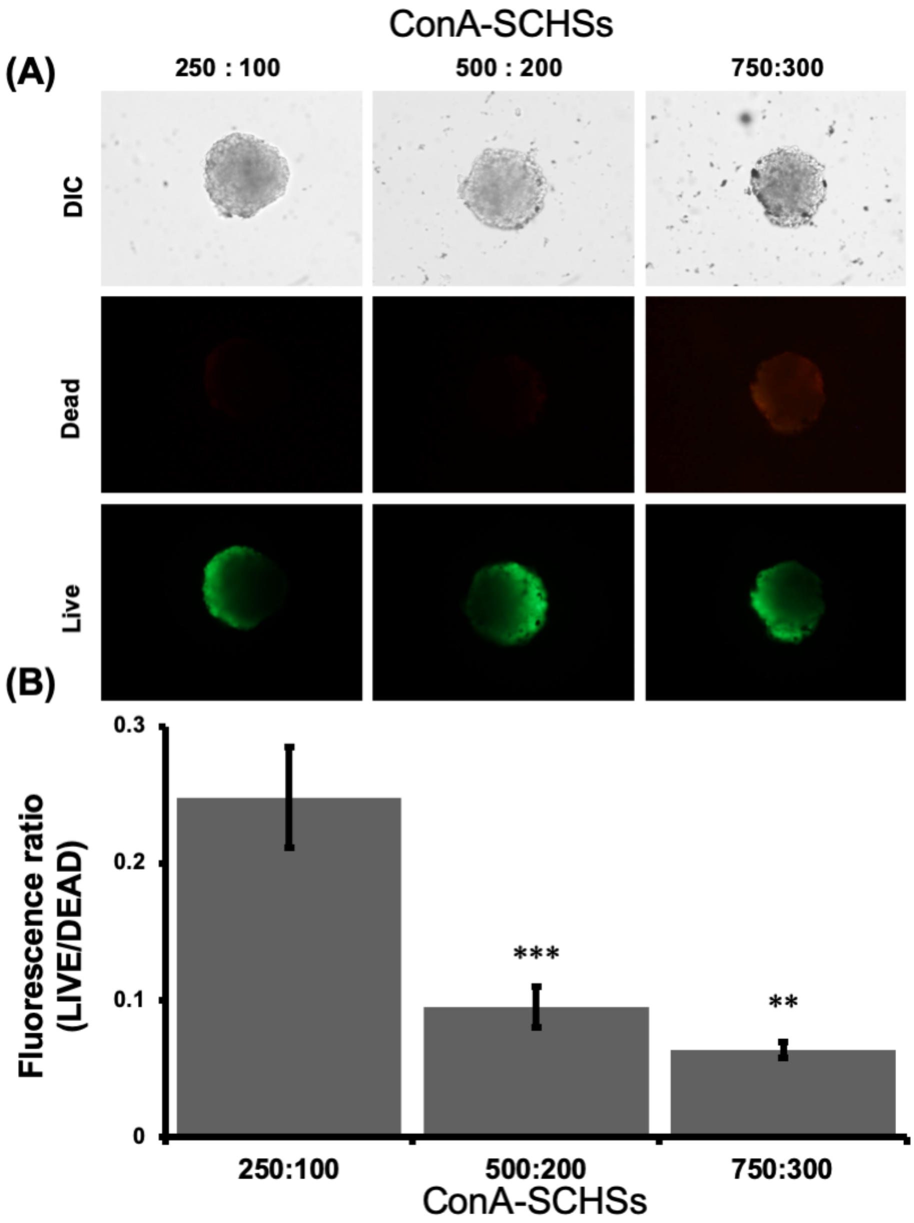

Figure S5. Optimization of the proportion of cells binding with ConA-SCHSs. (**A**) The proportion of the conjugation between ConA and SCHSs was identified and evaluated with LIVE/DEAD cell stain; (**B**) Quantification of the relative fluorescence reflects the ratio of live to dead cells at different proportions.
